# The entire organization of transcription units on the *Bacillus subtilis *genome

**DOI:** 10.1186/1471-2164-8-197

**Published:** 2007-06-28

**Authors:** Hirokazu Kobayashi, Joe Akitomi, Nobuyuki Fujii, Kazuo Kobayashi, Md Altaf-Ul-Amin, Ken Kurokawa, Naotake Ogasawara, Shigehiko Kanaya

**Affiliations:** 1 Department of Bioinformatics and Genomes, Graduate School of Information Sciences, Nara Institute of Science and Technology, 8916-5, Takayama, Ikoma, Nara 630-0192, Japan

## Abstract

**Background:**

In the post-genomic era, comprehension of cellular processes and systems requires global and non-targeted approaches to handle vast amounts of biological information.

**Results:**

The present study predicts transcription units (TUs) in *Bacillus subtilis*, based on an integrated approach involving DNA sequence and transcriptome analyses. First, co-expressed gene clusters are predicted by calculating the Pearson correlation coefficients of adjacent genes for all the genes in a series that are transcribed in the same direction with no intervening gene transcribed in the opposite direction. Transcription factor (TF) binding sites are then predicted by detecting statistically significant TF binding sequences on the genome using a position weight matrix. This matrix is a convenient way to identify sites that are more highly conserved than others in the entire genome because any sequence that differs from a consensus sequence has a lower score. We identify genes regulated by each of the TFs by comparing gene expression between wild-type and TF mutants using a one-sided test. By applying the integrated approach to 11 σ factors and 17 TFs of *B. subtilis*, we are able to identify fewer candidates for genes regulated by the TFs than were identified using any single approach, and also detect the known TUs efficiently.

**Conclusion:**

This integrated approach is, therefore, an efficient tool for narrowing searches for candidate genes regulated by TFs, identifying TUs, and estimating roles of the σ factors and TFs in cellular processes and functions of genes composing the TUs.

## Background

Recent progress in genome projects has generated a vast amount of nucleotide sequence data, and analyses of gene expression by global approaches have started to broaden our understanding of cell systems. As a useful model for systems biology and genomics, many studies use *Bacillus subtilis*, a spore-forming gram-positive bacterium whose genome sequence has been determined [1]. The ultimate goal of post-genome analysis is to specify transcriptional regulation in the entire genome. Computational algorithms to locate transcription units (TUs) have been developed based on analysis of signal sequences that are located at the boundaries of TUs from promoters to terminators, homologous gene pairs on other genomes, intergenic distance, functional categories, and gene clusters conserved among various species [2-7]. In the present study, a string of one or more genes co-transcribed is defined as a TU [4].

Identification of transcription factors (TFs) and their binding sites on their target genes is an important element of transcriptome analysis in the post-genome-sequencing era. Until now, various approaches have been taken to identify specific DNA-binding sites of TFs. DNA-binding specificities have traditionally been determined by experimental techniques such as DNase I footprinting and electromobility shift assay [8,9]. More recently, putative TF binding sites have been identified by computational techniques such as hidden Markov models (HMMs) [10] and position-weight matrices (PWMs) [11,12]. The PWM has one column for each position in the binding site and one row for each nucleotide. Each of the matrix elements is proportional to the relative frequency of the corresponding nucleotide at each position, and the score for a particular site is the sum of the matrix values for the sequence. Therefore, PWM is often used to predict nucleotide-protein binding sites and is used in the TRANSFAC database, which covers many known TFs and binding sites [13]. This approach is a convenient way to identify positions that are more highly conserved than others in a whole genome, because any sequence that differs from a consensus sequence has a lower score. The accuracy of detecting promoter sequences thus depends on the conservation of TF-binding sites.

We can now use complete genomic DNA sequences from several species and analyze massive amount of data on differential gene expression in microarray experiments [14]. Using microarrays in various conditions, we can obtain co-expression patterns for adjacent genes, which is an important property for determining transcription units.

In the present study, we identify the TUs in *B. subtilis *using a combination of (i) a bioinformatics approach, using PWM methods that identify TF-binding sites by detecting statistically significant TF-binding sequences on the genome; and (ii) two DNA microarray analyses, one to predict co-expressed gene clusters by calculating Pearson correlation coefficients of expression profiles for neighboring genes, and the other to determine genes regulated by each of the TFs in the units by comparing gene expression between wild-type and TF deletion mutants in the genome.

## Results

### The integrated strategy for TU prediction

**Figure 1:**
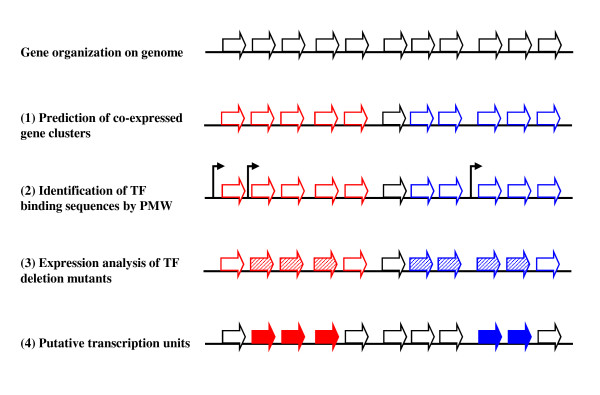
**Outline of procedure for elucidating TUs by integrated analyses**. (1) Co-expressed gene clusters predicted by correlating expression profiles between neighboring genes; (2) promoter (thin arrows) prediction by PWM as the start of a TU in the putative co-expressed gene clusters; (3) detection of significantly expressed genes (stripe thick arrows) by comparison between TF deletion mutant arrays and wild ones; (4) identification of genes composing putative TUs (filled thick arrows) by integration of the three analyses.

The procedures for elucidating TUs are outlined in Fig. 1. First, co-expressed gene clusters were determined by correlating expression profiles between neighboring genes transcribed in the same direction with no intervening gene transcribed in the opposite direction (Fig. 1(1)). Co-expression between neighboring genes was estimated using a t-test of the Pearson correlation coefficient. To predict co-expressed gene clusters on the *B. subtilis *genome, we used 98 DNA microarray data sets in 13 different time-series growth conditions. We then detected various sizes of co-expressed gene clusters and observed that most clusters consisted of four genes or less.

**Figure 2:**
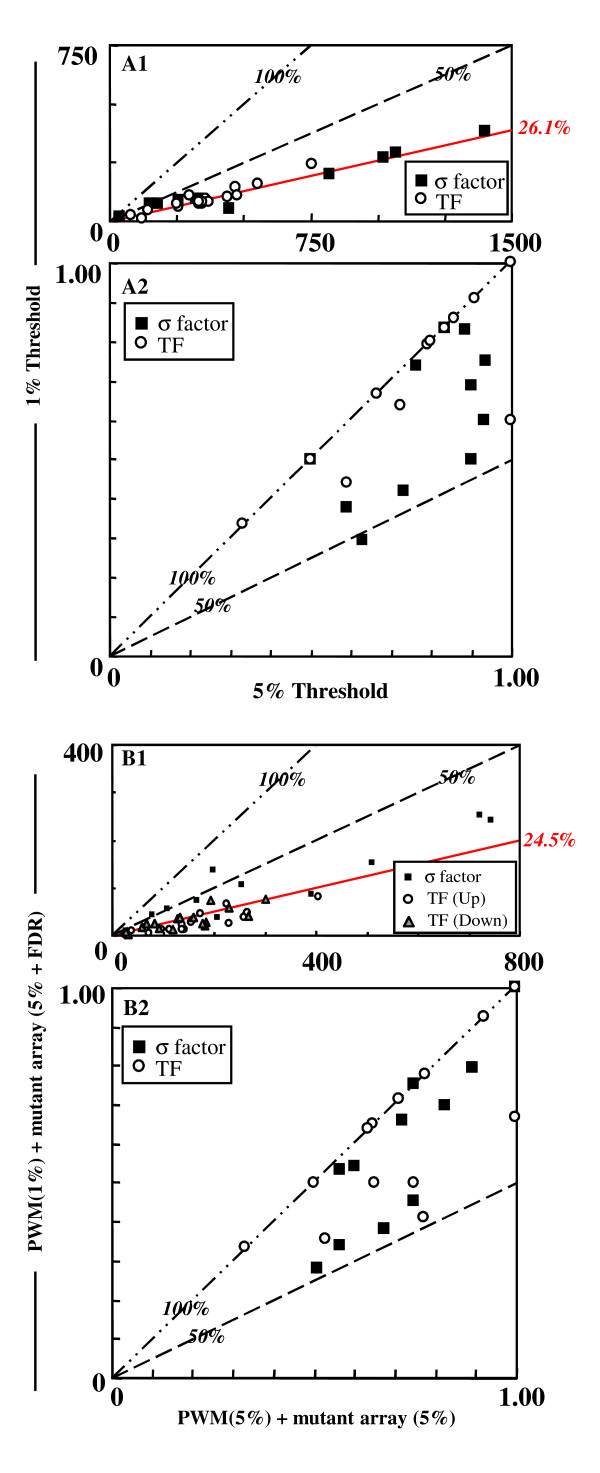
**Comparison of coverage and sensitivity in each of the TFs**. The coverage of promoters on the *B. subtilis *genome (A1), and the sensitivity of known promoter detection by PWM below the 1% threshold vs. that below 5% threshold (A2). The coverage of genes composing TUs on the *B. subtilis *genome (B1), and the sensitivity of detection of genes composing known TUs by this integrated analysis below the 5% threshold for PWM and the deletion mutant array without FDR vs. that below the 1% threshold for PWM and the deletion mutant array with FDR (B2).

Second, we regarded genes having promoters predicted by PWM as the start of the TUs (Fig. 1(2)). There are at least 18 different σ factors that direct RNA polymerase, and a large number of sequence-specific DNA binding proteins that play various roles of controlling gene expression, as promoter activators or repressors in *B. subtilis *[15,16]. We then examined the TF-binding promoter sequences of 11 σ factors except σA, which are known to possess multiple *cis *elements, and 17 TFs within 300 bp upstream of an open reading frame for all 4,219 genes of *B. subtilis *by PWM, and found putative promoters regulated by each TF below the thresholds. Figure 2A shows a comparison of coverage (Fig. 2A1) and sensitivity (Fig. 2A2) between the 1% and 5% thresholds. We were able to narrow down the candidates for TF-binding sites to 26.1% of the candidates (i.e. from 431 to 110 sites) when we changed the threshold from 5% to 1% (Fig. 2A1). On the 5% threshold, we identified an average of 78% known promoters, and an average of 69% promoters on the threshold of 1% (Fig. 2A2). Thus, the average difference of detecting known promoters is 9%, corresponding to 3.5 promoters, by changing the threshold from 5% to 1%. Furthermore, in most TFs we could efficiently narrow down candidates for the TF binding site, and found that the number of known promoter sites detected below each of the thresholds hardly changed. Therefore, we took these PWM analyses at the threshold of 1%.

Third, we derived significant expression change data from TF deletion mutant microarray data to identify genes regulated by each of the TFs (Fig. 1(3)). In these analyses, we applied a one-sided test to examine genes whose expression changed significantly in the normalized microarray data, and found candidate up-regulated genes for 28 TFs, including 11 σ factors and candidate down-regulated genes for 17 TFs. Furthermore, we used the false discovery rate (FDR) procedure to remove false-positive data from the candidates of significant expression change data and narrow the candidates for genes regulated by each of the TFs [17].

We then integrated these analyses, and present a comparison of coverage (Fig. 2B1) and sensitivity (Fig. 2B2) between two integrated conditions (i.e. a 5% threshold at PWM and a 5% threshold in the deleted mutant array without FDR, and a 1% threshold at PWM and a 5% threshold in the deleted mutant array with FDR). We were able to narrow down the candidate genes composing TUs at the 5% PWM without FDR to 24.5% of the candidates (i.e. from 194 to 50 genes) when we changed the condition from the PWM 5% threshold without FDR to the PWM 1% threshold with FDR, with 87.1% of genes detected at 5% PWM without FDR also being detected at 1% PWM with FDR. Thus, the candidates can be effectively narrowed without remarkable loss of regulation-known genes under the condition of 1% PWM with FDR. The σL, PerR, and PurR TUs were efficiently detected. Regarding the σL TUs in particular, we could narrow down the 63 TU candidates for the PWM 5% threshold to 9 candidates for the PWM 1% threshold with FDR control without any loss of sensitivity. The detected TUs for the 1% PWM with FDR and known TUs regulated by each of the TFs are listed in Additional file 1.

### Organization of TUs in *B. subtilis*

**Figure 3:**
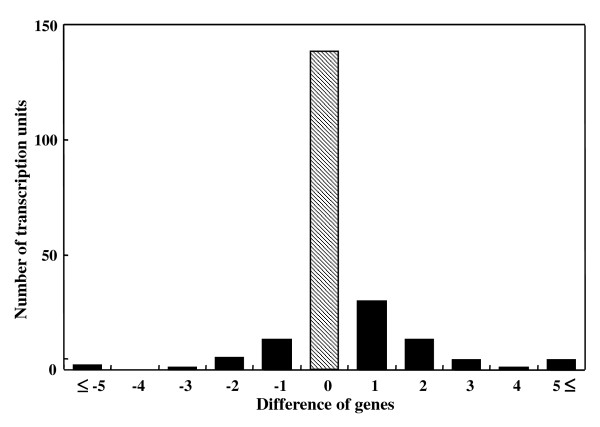
**Comparing TUs predicted in the present study with known *B. subtilis *operons, which start with the same promoters as the TUs**. 65.6% of the known TUs matched the predicted units (i.e., 139 predicted TUs in the 212 known *B. subtilis *operons); when we included TUs lacking or gaining one gene of known TUs, this figure rose to 85.8%. The x-axis indicates difference in gene number between predicted transcriptions units and known *B. subtilis *operons, and the y-axis indicates the number of predicted TUs.

The difference between TUs predicted in the present study and known operons in *B. subtilis *indicates that most of the predicted TUs are consistent with those reported (Fig. 3). Consequently, the entire TU map on *B. subtilis *genome can be estimated on the basis of the predicted TUs. At the 1% PWM with FDR, we can pick 2,183 genes composing 892 TUs, which include known operons, from the complete *B. subtilis *genome. The average size of the polycistronic transcription unit is 3.71 genes, which is comparable in size to those in *Staphylococcus aureus *(3.47 genes) [5] and in *E. coli *K12 (3.41 genes) [4]. Distribution of the TUs to the number of genes is almost identical between *B. subtilis *and *S. aureus *(Additional file 2) [5]. Thus the operon organization of those two gram-positive bacteria are fundamentally identical and are approximated by power-law equations, where the correlation of the double logarithm linear relation between the numbers of genes and of TUs composed by the genes is -0.98 in *S. aureus *and -0.97 in *B. subtilis*.

## Discussion

In the present study, we identified various sizes of TUs regulated by each TF and detected gene clusters consisting of part of well-known operons (*yabPQ *regulated by σE and *divIC-yabR *regulated by σX in *yabMNOPQ-divIC-yabR *operon, *nasDEF *regulated by GlnR in *nasBCDEF *operon, *yjmEFGHIJ *regulated by σE in *yjmABCDEFGHIJ *operon, *spoVE-murG *regulated by σE in *murE-mraY-murD-spoVE-murG-murB-divIB-ylxWX-sbp *operon, *xynB *regulated by XylR in *ynaJ-xynB *operon and *yoxB-yoaA *regulated by σB in *yoxCB-yoaA *operon mentioned in Additional file 1). They are known to be regulated by internal promoters and to constitute functional components [18], for instance, *yabPQ *regulated by σE that plays an important role in synthesis of the spore cortex and coat [19], and *divIC-yabR *regulated by σX which is essential for the initiation of vegetative septum formation [20,21] in *yabMNOPQ-divIC-yabR *operon. Therefore, these gene clusters separated by internal promoters tend to be functional units.

**Figure 4:**
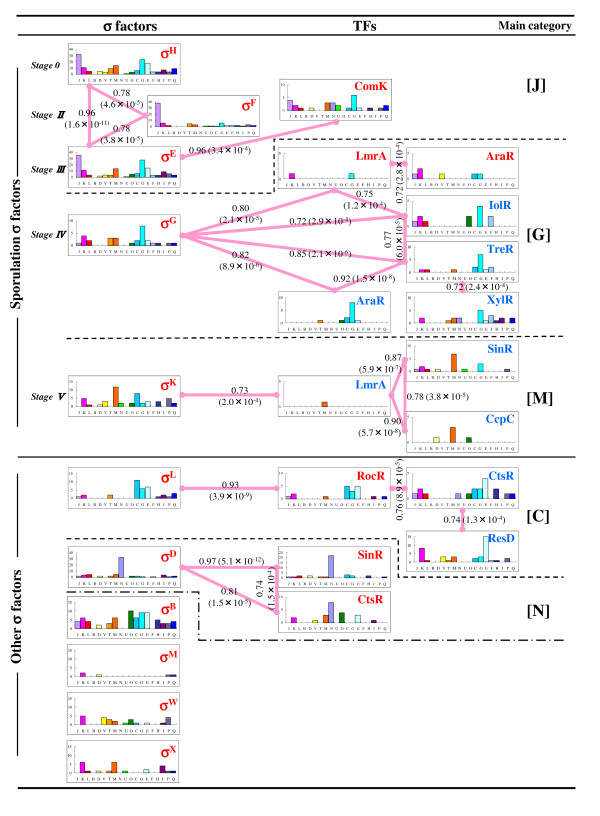
**Clustering of TFs based on the functional similarity of genes composing the TUs**. For clustering of TFs, Pearson correlation coefficients among TFs were calculated using the frequencies of genes belonging to each of the COG categories. We regarded groups of TFs that have correlation coefficients above 0.70 (solid lines) as the clusters. Pearson correlation coefficients and P-values in the parentheses are represented on the solid lines. The main category represents the COG category into which the most genes regulated by each of the TFs in the clusters are classified. One-letter abbreviations use used for the functional categories: J, translation, ribosomal structure and biogenesis; K, transcription; L, DNA replication, recombination and repair; D, cell division and chromosome partitioning; O, posttranslational modification and protein turnover, chaperones; M, cell envelope biogenesis and outer membrane; N, cell motility and secretion; P, inorganic ion transport and metabolism; T, signal transduction mechanisms; C, energy production and conversion; G, carbohydrate transport and metabolism; E, amino acid transport and metabolism; F, nucleotide transport and metabolism; H, coenzyme metabolism; I, lipid metabolism; Q, secondary metabolite biosynthesis, transport and catabolism. All data were identified under PWM with 1% threshold and FDR control.

Using the TU data, we examined the transcriptional regulation of genes by 11 σ factors whose promoter sequences have been characterized. The properties of individual σ factors are as follows: five σ factors (σE, σF, σG, σH, σK) regulate sporulation through morphological stages that involve the conversion of the growing cell to a two-cell sporangium, which ultimately proceeds to a single spore; σB mediates the general stress response, and more than 150 protein-coding genes for general stress belong to the σB regulon [22]; σL mediates cold-shock adaptation and regulation of the acetoin catabolic pathway [23]; σD regulates flagellar synthesis, motility, and chemotaxis [24]; σM mediates salt resistance [25]; and σX and σW play modulatory roles in extracytoplasmic function [26]. All the regulative relations of the 11 σ factors and 17 TFs to targeted genes are listed in Table S1, making it possible to characterize individual σ factors according to the genes they target. Therefore, we classified genes belonging to each of the TUs into 19 COG (clusters of orthologous groups of proteins) functional categories [27] for estimating the general roles of the σ factors and TFs in cellular processes (Fig. 4).

The similarity of the roles in cellular process between individual TFs was estimated using Pearson correlation coefficients for the number of genes belonging to each of the COG categories (Fig. 4). The five σ factors associated with regulation of the sporulation process can be classified into three groups corresponding to the sporulation process Stage 0-III (σH, σF and σE) characterized by category [J; translation, ribosomal structure and biogenesis], Stage IV (σG) characterized by the category [G; carbohydrate transport and metabolism], and Stage V (σK) characterized by category [M; cell envelope biogenesis and outer membrane]. Gene expression under the σG control occurs in the prespore, and the main functions are to protect the spore from several hazardous conditions, high osmotic pressure [28], UV radiation and dry heat [29], and to prepare the spore for germination and outgrowth [30]. In this process, σG regulates carbohydrate content in the cell, for example, by activating expression of the glucose dehydrogenase operon [31], controlling metabolism of the tricarboxylic acid cycle [32] and glucose uptake [33]. σK is synthesized and becomes active in the mother cell, and directs formation of the spore coat and spore maturation [30]. Therefore, these previous experimental studies are consistent with the present results. Moreover, we can observe that each TF in a cluster has one of the frequently detected functional categories (Fig. 4). The AraR protein is well known as a negative regulator of the L-arabinose metabolic operon [34], and most of the genes negatively regulated by AraR belong to [G] (Fig. 4). Almost all the genes up-regulated by SinR are in category [N], which consists of proteins controlling cell motility and secretion, while the down-regulated genes belong to category [M], which consists of proteins operating cell-wall and membrane biogenesis (Fig. 4). ComK synthesis is regulated by a series of reactions that involve quorum sensing; SinR is one of the activators in this cascade, acting negatively on *rok *transcription [35], and is known to be a potent repressor of biofilm formation [36]. Thus, the analysis presented here agrees well with previous experimental data and enables us to assess the roles of the σ factors and TFs in cellular processes.

**Figure 5:**
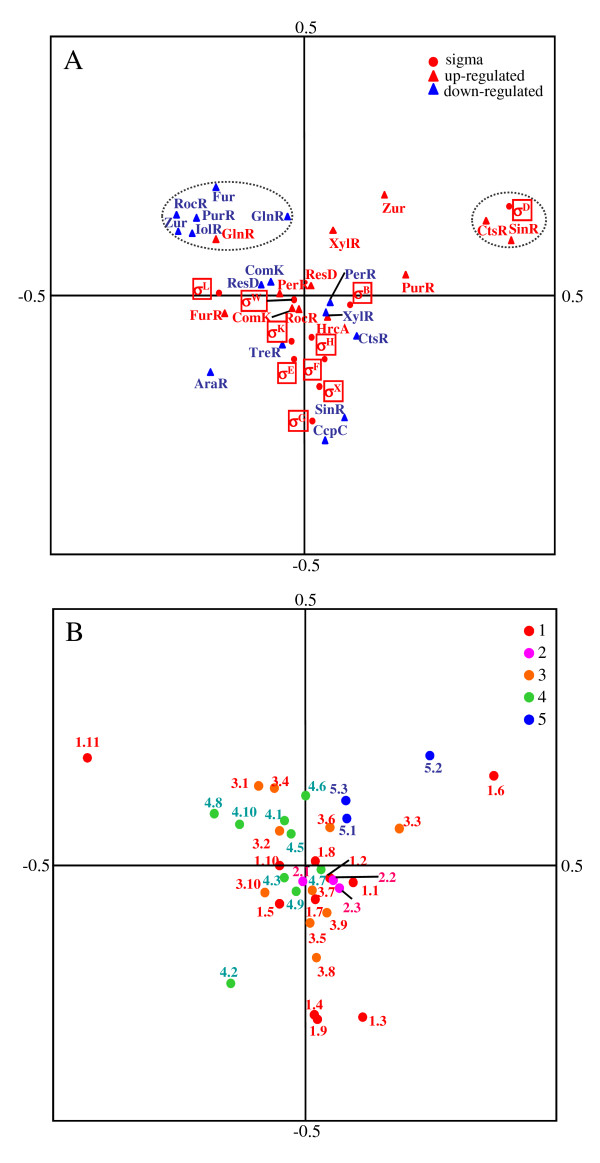
**Score plots for PCA of TFs and functional categories**. (A) Score plot based on frequencies of TF functions. (B) Score plot based on frequencies of functional classification of the *B. subtilis *protein-encoding genes. Abbreviations for the functional categories: 1, cell envelope and cellular processes [1.1, adaptation to atypical conditions; 1.2, cell division; 1.3, cell wall; 1.4, germination; 1.5, membrane bioenergetics (electron transport chain and ATP synthase); 1.6, motility and chemotaxis; 1.7, protein secretion; 1.8, sensors (signal transduction); 1.9, sporulation; 1.10, transformation/competence; 1.11, transport/binding proteins and lipoproteins]; 2, cytochromes [2.1, cytoplasmic; 2.2, membrane-bound; 2.3, other cytochromes]; 3, information pathway [3.1, competence regulatory; 3.2, detoxification; 3.3, DNA packaging and segregation; 3.4, DNA replication; 3.5, DNA restriction/modification, repair and recombination; 3.6, protein folding; 3.7, protein modification; 3.8, protein synthesis; 3.9, RNA modification; 3.10, RNA synthesis]; 4, intermediary metabolism [4.1, antibiotic production; 4.2, carbohydrates and related molecules; 4.3, degradation; 4.4, general function prediction; 4.5, metabolism of amino acids and related molecules; 4.6, metabolism of coenzymes and prosthetic groups; 4.7, metabolism of lipids; 4.8, metabolism of nucleotides and nucleic acids ; 4.9, metabolism of phosphate; 4.10, metabolism of sulfur]; 5, other functions [5.1, antibiotic production; 5.2, phage-related functions; 5.3, transposon and insertion elements].

In addition, the genes targeted by σ factors and TFs are classified into 36 categories based on functional classification of the *B. subtilis *protein-encoding genes [37] to examine the role similarities among them based on *B. subtilis*-specific gene functions such as the endospore-formation process. We then show the projection of σ factors and TFs in the largest two principal components (Fig. 5A) and factor loadings of individual categories, indicating the contribution of the category frequencies to the two principal components based on the frequencies of the 36 categories (Fig. 5B). We observe a small cluster composed of σD, CtsR and SinR (a broken line circle in Fig. 5A), which is consistent with the result in Fig. 4. Here, σD is the σ 28-form subunit of RNA polymerase, and many σD-dependent genes are known to be necessary for flagellar synthesis and motility functions [38]. In addition, CtsR controls the expression of heat-shock proteins that are required for stress tolerance and growth at high temperature [39], and play essential roles in competence development and motility [40]; SinR also regulates the development of genetic competence and motility [41]. Thus, the roles of these three TFs in cellular processes are associated with motility, and those are plotted in the same region of the cluster characterized by category [1.6, motility and chemotaxis] (Fig. 5A and 5B). This result shows that roles of TFs can be estimated by the principal component analysis (PCA) based on comprehensive searches for functions of gene composing these TUs.

It can also be seen in another cluster composed of Fur, Zur, IolR, PurR, RocR, and GlnR (a broken line circle in Fig. 5A). Fur and Zur regulate the expression of ABC transporters and both TFs control iron and zinc uptake and homeostasis pathways in response to available metals [42,43]. IolR and PurR also control transport systems. IolR regulates genes encoding inositol transporters and inositol uptake [44], while PurR regulates purine transport, metabolism, and biosynthetic pathways [45]. In this cluster, RocR and GlnR relate to controlling nitrogen sources: RocR controls arginine catabolism [46] and the arginase pathway in which arginine is converted to glutamate [47], while GlnR regulates responses to nitrogen availability, such as nitrogen metabolism [48] and assimilation [49].

Based on these previous studies, this result shows that we can cluster together homeostatic regulation TFs (Fig. 5A). Moreover, σ factors that regulate sporulation (σE, σF, σG, σH, σK) tend to exist near the y-axis in the region of lower first-principal component (PC1) values with negative PC2 values, and TreR, SinR, and CcpC are also plotted near the σ factors (Fig. 5A). TreR regulates trehalose as the sole carbon and energy source of *B. subtilis *during spore outgrowth [50], while SinR controls regulatory genes involved in the early stages of sporulation [51]. Thus, sporulation-related TFs tend to have lower PC1 values and negative PC2 values, which may be evidence that category [1.9; sporulation] and [1.4; germination] are plotted in the area (Fig. 5B). Therefore, CcpC is known to be a regulator of the tricarboxylic acid cycle genes [52], but may also have a function in regulating sporulation genes.

## Conclusion

This study presented the new approach to TU prediction in the bacterial whole genome using integrated analysis of microarray and DNA sequence data, and we efficiently detected genes composing TUs in *B. subtilis *genome. The results demonstrate that the combined approach is very useful for identifying unknown TUs in the genome, and also detecting internal operons in the known operons. This methodology should contribute to studies of predicting TU locations in the bacterial genome and estimating roles of TFs.

## Methods

### Bacterial strains, medium, growth conditions and RNA extraction

For expression profile analyses, *B. subtilis *168 was grown in 13 different time-series growth conditions: anaerobic growth; competent medium; cold-shock experiments; DSM medium; DGG medium; glucose-limited medium; heat-shock experiment; LB medium; minimum-glucose medium; sodium-shock conditions; phosphate-starvation medium; and SOS stress experiments. For TF deletion mutant analyses, TF deletion mutants were grown at 37°C in different medium conditions: LB medium for *sigB, L, M, W, X, araR, ctsR, hrcA, iolR, lmrA, rocR, sinR, xylR *deletion mutants; LB medium with trace elements for *fur *and *perR *deletion mutants; DSM medium for *sigD, E, F, G, H, K, treR *deletion mutants; DSM medium with 2% Gln and 5% glucose for, respectively, *glnR *and *resD *deletion mutants; MC medium for the *comK *deletion mutant; MGM medium for the *ccpC *deletion mutant; and MGM with adenine and guanine for the *purR *deletion mutant. Cells were harvested by centrifugation at 1,000 g after adding the RNA-protecting Bacteria Reagent (Qiagen), and then stored at -80°C. Two independent samplings were performed. RNA was isolated using the RNA protectant, RNeasy Mini and RNase-free DNase kits (Qiagen) according to the manufacturer's instructions and stored at -80°C. Genomic contamination was estimated by gel electrophoresis.

### Labeling

For each labeling reaction, a total of 15 μg of RNA was used. First-strand cDNA synthesis was primed with 1.2-μg random primers (Invitrogen) in nuclease-free water (total volume: 31 μl) by heating at 70°C for 10 min and incubating at 25°C for an additional 10 min. Reverse transcription was performed by SuperScript III (Invitrogen) in reverse transcription buffer [1 × first-strand buffer, 10 mM DTT] in the presence of 5 mM dATP, 5 mM dUTP, 5 mM dCTP, 0.25 mM dTTP, and 0.25 mM AA-dUTP. Three amino-allyl-labeled nucleotides were incorporated into the cDNA. The reactions were incubated at 25°C for 10 min, 37°C for 60 min, 42°C overnight, and quenched by heating at 70°C for 10 min.

The RNA template was hydrolyzed by adding 20 μl of 1N NaOH followed by heating at 65°C for 30 min. Reactions were neutralized with 20 μl of 1N HCl. cDNA was purified using a CyScribe GFX Purification Kit (GE Healthcare) according to the manufacturer's directions. NHS ester forms of Cy3 and Cy5 dyes were added to the cDNA solution and incubated for 4 hr. Coupling reactions were quenched by the addition of 15 μl of 4 M hydroxylamine and incubated at room temperature for 15 min in the dark. Labeled cDNA was purified using the CyScribe GFX Purification Kit again.

### Hybridization and spot detection

Prehybridization of the array slides was performed for 3 hr in filtered prehybridization solution [25% formamide, 5× SSC, 10 mg BSA (fraction V), 0.1% SDS] at 42°C. Slides were briefly washed in milliQ water and 80% ethanol and dried by centrifugation at 1,000 g for 5 min. Hybridization of the probe was performed using hybridization solution (25% formamide, 5× SSC, 0.1% SDS, 0.1 μg poly(A), 1 × Denhardt's solution and 100 pmol Cy3 and Cy5 combined probe). The hybridization solution containing the Cy-dye-labeled cDNA was heated to 95°C for 3 min and hybridization was performed in an Advalytix hybridization machine (ArrayBooster) at 42°C for 16 hr. After hybridization, the slides were washed and dried by centrifugation at 1,000 g for 5 min and then analyzed using a Fuji FLA-8000 scanner and Array Gauge ver.2.0 software (Fuji Film).

### Normalization in microarray experiments

Gene expression levels are evaluated by scanning the fluorescence intensity for each spot, and there is usually some experimental variation that occurs in every microarray experiment. It is, therefore, important to minimize experimental variation, and although several methods of microarray normalization have been developed [53,54], there are usually some false-positive data arising when analyzing gene expression data collected via microarrays.

Normalization of the logarithmic ratio of expression intensity between target (R_i_) and control (G_i_) experiments was carried out based on MA plots [55], which can show the intensity-dependent ratio of raw microarray data using TREBAX [56]. The plots differed in the axes used. The MA plot used M_i _(log_10 _(R_i_/G_i_)) as the y-axis and A_i _(log_10 _RiGi
 MathType@MTEF@5@5@+=feaafiart1ev1aaatCvAUfKttLearuWrP9MDH5MBPbIqV92AaeXatLxBI9gBaebbnrfifHhDYfgasaacH8akY=wiFfYdH8Gipec8Eeeu0xXdbba9frFj0=OqFfea0dXdd9vqai=hGuQ8kuc9pgc9s8qqaq=dirpe0xb9q8qiLsFr0=vr0=vr0dc8meaabaqaciaacaGaaeqabaqabeGadaaakeaadaGcaaqaaiabbkfasnaaBaaaleaacqqGPbqAaeqaaOGaee4raC0aaSbaaSqaaiabbMgaPbqabaaabeaaaaa@3210@) as the x-axis. By plotting values of A_i _on the abscissa and M_i _on the ordinate of a coordinate system, it was possible to evaluate the bias error with respect to the average logarithmic intensities. The normalized log ratio M"_i _was estimated as the difference between M_i _and baseline M'_i_. Here, using the relation between M_i _and A_i _(M_i _= *f *(A_i_) + *ε*_*i*_, where *ε*_*i *_is the difference between M_i _and *f *(A_i_) for gene i) for the MA plot, the baseline for the *i*th gene was estimated by M'_I _= *f *(A_i_). Genes whose signal intensity was regarded as zero were eliminated from the present analysis. With this methodology, it is assumed that there was no large error due to expression intensity in the majority of the spots.

### Prediction of co-expressed gene clusters

Co-expressed gene clusters were predicted based on expression profiles and genomic locations. The expression profile of the *i*th position gene is represented by vector x_*i*_, consisting of logarithmic ratios for microarray experiments. The algorithm for predicting co-expressed gene clusters is as follows: we selected a series of genes transcribed in the same direction with no intervening gene transcribed in the opposite direction. The genes were denoted g_1_, g_2_, ... g_i_, ..., g_M _from their 5' to 3' termini. Here, g_i _and g_i+1 _(i = 1, 2, ..., M-1) are adjacent genes on the same DNA strand. First, Pearson correlation coefficients (r_st_) were estimated for all pairs of vectors *x*_*s *_and *x*_*t *_(s = 1, 2, ..., M; t = 1, 2, ..., M). Second, a pair of genes was assigned to a candidate group. Gene g_s _always belonged to group G_s _(s = 1, 2, ..., M). All the genes g_s+1_, g_s+2_, ..., g_s+Ts_, whose correlations r_s(s+1)_, r_s(s+2)_, ..., r_sTs _were statistically significant in a t-test at the 5% significance level, were classified into G_s_. In the same manner, all the genes g_s-1_, g_s-2_, ..., g_s-Us_, whose correlations r_s(s-1)_, r_s(s-2)_, ..., r_sUs _were statistically significant in a t-test at the 5% significance level, were also classified into G_s_. Thus, altogether T_s _+ U_s _+ 1 genes were classified into group G_s_. Finally, all members of group G_s _(s = 1, 2, ..., M) were compared. We counted the number of groups consisting of identical members among G_s _(s = 1, 2, ..., M) and selected the group having the highest count as the first co-expressed gene cluster T_1_. After excluding the T_1 _genes from all the groups (g_1 _to g_M_), we selected the next-highest identical group as the next co-expressed gene cluster T_2_. This procedure was carried on until the number of members in T_v _was zero, or until all positions j (j = 1, 2, ..., M) were occupied by genes belonging to T_v_.

### Identification of promoter sequences by PWM

DNA sequences recognized by TFs consist of consensus regions. We searched for sequences highly homologous to those known to be recognized by TFs using PWM. First, we prepared datasets of training sequences consisting of experimentally determined promoters from DBTBS [57] and "*B. subtilis *and Its Closest Relatives: from Genes to Cells" [37], which were aligned on the basis of their consensus regions. PWMs for individual TFs were constructed by the frequencies *F*_*Ak*_, *F*_*Tk*_, *F*_*Gk*_, and *F*_*Ck *_of the four nucleotides (A, T, G, C) in the *k*th position, including the consensus regions and the five bases upstream and downstream. We determined the score by multiplying all the frequencies corresponding to a given sequence. Second, the thresholds for the binding sites were determined as follows: 4,000 DNA sequences each comprising 300 nucleotides were generated randomly based on the GC content of *B. subtilis*. The threshold was defined by the value below which the lowest 95% of the maximum scores in individual DNA sequences were excluded. Third, within the 300-nucleotide sequence upstream of the protein-coding region, individual TF binding sites were predicted by the maximum PWM score above the threshold because about 95% of TF binding sites were known to exist in these regions. We chose optimal matrices for each random sequence, and regarded sequences that exceeded the threshold as being regulated by the TF. Therefore, we used these sequences to search for other sequences highly similar to those recognized by TFs. This was done by calculating scores for the partial sequences in the stretch of 300 nucleotides upstream of the protein-coding regions of all *B. subtilis *genes. Sequences whose scores exceeded a threshold were regarded as TF-binding sites.

### Expression analysis of TF deletion mutants of *B. subtilis*

The normalized fluorescence intensity data were analyzed using a one-sided test to compare the results of the deletion mutant to the control samples, and genes whose expression exceeded the threshold were regarded as TF-regulated genes. In lower one-sided tests, we considered genes of decreased expression as being up-regulated by the TF, whereas genes of increased expression were considered as down-regulated by the TF in upper one-sided tests.

### False discovery rate

For separating inactive genes from those that were deemed active in the expression analysis of TF deletion mutants, we used the false-discovery rate, an alternative approach to multiple testing [58]. On the assumption that we conducted *m *multiple tests, the null hypothesis that each gene is differentially expressed is true for *m*_0 _tests, and the alternative hypothesis is true for *m*_*1 *_(= *m *- *m*_0_). Among the *m*_0 _null hypotheses, U hypotheses were declared false-negative and V (= *m*_0 _- U) hypotheses were declared true-positive. Among the *m*_*1 *_alternative hypotheses, T hypotheses were called true-negative and S (= *m*_*1 *_- T) hypotheses were called false-positive. R (= V + S) is the total number of hypotheses rejected and an observable random variable. The FDR was defined as *π*_0 _= P (R > 0) E (V/R | R > 0), and we thus regarded R (1 - *π*_0_) as the number of true active genes.

## Authors' contributions

HK designed and carried out the statistical studies, and drafted the manuscript. JA and NF contributed bioinformatics and statistical studies for sequence analyses. KK and NO designed and conducted the microarray experiments. AA provided bioinformatics support of microarray analyses. KEN and SK contributed substantially to manuscript preparation and editing. NO and SK designed and oversaw the project. All authors read and approved the final manuscript.

## Supplementary Material

Additional file 1 Supplementary Table 1. Transcription units regulated by the TFs in the *Bacillus subtilis *genome. Click here for file

Additional file 2 Supplementary Figure 1. The relationship between the number of TUs detected in the present study and the number of genes composing the TUs. Click here for file
