# Supplementary material for: The entire organization of transcription units on the Bacillus subtilis genome
Source: BMC Genomics. 2007 Jun 28;8:197. doi: 10.1186/1471-2164-8-197 (PMC1925097; doi:10.1186/1471-2164-8-197)
Supplement: Additional file 1 — Supplementary Table 1. Transcription units regulated by the TFs in the Bacillus subtilis genome. [file 1471-2164-8-197-S1.pdf]

TF[a]		Identified transcption units[b]
**σB** (246)	**U**	***aldY***, *aprE*, ***bmrU***, ***bofCcsbXyrbE***, ***cdd***, *citCZ*, ***clpP***, ***csbA***, ***csbB***, ***ctc***, *dapAymfA*, *dnaJKgrpEhrcAhemNlepA*, ***dps***, *glgPA*, *glnRA*, ***gsiBydbBC***, ***gspA***, ***gtaB***, *iolIH*, ***katX***, ***nadE***, ***opuE***, *paiB*, *panBbirA*, ***phoRP***, *pksIJKLMNP*, *ppsCB*, ***rsbVWsigBrsbX*** *ydcFGH*, ***sigAdnaGyqxD***, ***spoVG***, *sunAyolF*, ***trxA***, *uvrB*, *uvrC*, *xkdIJKMNO*, *xkdRS*, *yaaH*, ***yaaI***, *yabNOPQ*, ***yacHIclpCsmsyacKLM***, ***ybyB***, ***ycbP***, *ycdD*, *ycdFG*, *yckC*, ***ycnH***, ***ydaB***, ***ydaC***, ***ydaG***, ***ydaJ***, *ydaKLMN*, ***ydaP***, ***ydaST***, ***ydbD***, ***ydbP***, *ydcOPQR*, *yddGH*, *ydfB*, ***ydfO***, ***ydgC***, ***ydhK***, *yebE*, ***yerD***, *yfhD*, ***yfhE***, ***yfhF***, ***yfhJKLM***, ***yfkJIH***, ***yflA***, ***yflS***, *yflT*, ***ygxB***, ***yhaR***, ***yhaUTS***, ***yhcM***, ***yhdF***, ***yhdN***, *yhdP*, ***yheK***, *yhfP*, ***yhxD***, ***yjbCD***, *yjcE*, ***yjfByjgB***, ***yjgCD***, *ykgA*, ***ykgBA***, *ykuL*, *ykzA*, *yllBylxAftsL*, *ynfC*, ***yocB***, ***yocK***, *yosCB*, ***yotK***, ***yoxByoaA***, *ypbR*, *yphB*, *yphPilvDypgRQbsaA*, ***ypuCB***, ***ypuD***, *yqeGF*, *yqfDC*, ***yqgZ***, ***yqhA***, ***yqhB***, ***yqhQP***, *yqiSR*, ***yqxL***, *yrhK*, ***yrvD***, ***yrvIrelA***, ***ytaB***, ***ytkL***, ***ytxJHG***, *yueKJIH*, ***yunG***, *yurMN*, ***yuzA***, *yvaA*, ***yvaK***, ***yvbG***, ***yvgN***, ***yvgO***, ***yvgW***, ***yvgZ***, ***yvrE***, ***yvyD***, ***ywdD***, ***ywjC***, ***ywlE***, ***ywmE***, ***ywmG***, *ywnJspoIIQ*, ***ywtCBAywsC***, *ywtG*, ***yxbG***, ***yxcC***, ***yxiSkatB***, *yxjHG*, ***yxkO***, ***yxnA***, ***yxzFyxlJ***, ***yycD***, ***yycE***
**σD** (154)	**U**	***cheV***, *cotN*, ***degR***, ***dltABCDE***, *dps*, ***epr***, *etfABysiBA*, ***fliIJylxFfliKylxGflgEfliLMYcheYfliZPQRflhBAF-ylxHcheBAWCDsigDylx*** *LrpsB*, ***fliTSDyvyC***, ***hag, lytCBA***, ***lytD***, ***mcpCykwC***, ***motBA***, ***nucAtlpC***, *phrIyddM*, ***sigA***, ***tlpBmcpAtlpAmcpB***, *xkdA*, *ybdO*, ***yfmTS***, ***yhdD***, *yhfUV*, ***yjbJ***, ***yjcPQ*** *RSyjdA*, ***yjfB***, *ykoVW*, ***ylqB***, *ylxPrbfA*, ***yoaH***, *yocJ*, *yokFE*, *yolBA*, *yomK*, *yonR*, *yopJI*, *yoqLKJ*, *yorZYXWV-mtbPyorTSRQPONMLKJIHGFEDCBAyoqZY*, *yosRQPONMLKJIHG*, ***yscB***, *yvaQ*, *yviFE* ***flgLKyvyGflgMyvyFcomFC***, *yvzD*, *ywcHG*, ***yxkC***
**σE** (315)	**U**	*aspShisS*, *citMyflN*, *comPX*, *cotAgabP*, ***cotE***, *cotZYXW*, ***ctaA***, ***cwlJ***, *enopgmtpipgkgapyvbQ*, *fruRBA*, *gerBABBBC*, ***gerM***, *glcTptsG*, ***glgPADCB***, *glnHMP*, *homyutH*, ***mblspoIIIDusd***, ***nucB***, *odhBA*, *opuAAABAC*, *opuCDCCCBCA*, ***phoRP***, ***prkA***, *purA*, *recRyaaL* ***bofA*** *rrnA*, *rplBrpsSrplVrpsCrplPrpmCrpsQrplNXErpsNHrplFRrpsErpmDrplOsecYadkmapinfA-rpmJrpsMKrpoArplQybxAybaEFtruArplMrpsIybaJK* ***cwlD***, *rpsPylqCDEtrmDrplS*, ***sigG***, ***spmBAdacB***, ***spoIID***, ***spoIIIAHIIIAGIIIAFIIIAEIIIADIIIACIIIABIIIAA***, ***spoIIM***, ***spoIVA***, ***spoIVCA***, ***spoIVCB***, ***spoIVFBI***, ***spoVB***, ***spoVD***, ***spoVEmurG***, ***spoVK***, ***spoVM***, ***spoVR***, *spsKJIGF*, *sspEygaB*, *tig*, *tkt*, *trePARyfkO*, *uppglyA*, ***valSysxE***, ***yaaH***, *yabGvegsspF*, ***yabMNO***, ***yabPQ***, *yacD*, *ybaL*, ***ybaN***, *ybbCDEF*, ***ycgFG***, ***ydcA***, ***ydcC*** *dal*, *ydhF* ***phoB***, *ydjDE*, ***yfhS***, *yfjS*, *yfkRQ*, *yhaUTS*, ***yhaX***, ***yhbH***, *yhcOP*, *yheBA*, *yheM*, *yhjM*, *yisCDEFGH*, ***yisO***, *yjbFG*, ***yjbX***, ***yjmEFGHIJ***, ***yknT***, *yknU*, *yknWXYZ*, *yktA*, ***ykvI***, *ykvS*, ***ykvUV***, *ylaJ*, *ylaK*, *ylbBCD*, ***ylbJ***, *ylbKL*, *ylbQyllA*, *yndL*, *yneNtlp*, ***yngDEFGHI***, *yoaU*, ***yoaW***, *yotK*, *ypbF*, *yphB*, ***ypjB***, *yptA*, *yqeVUTdnaJKgrpEhrcAhemNlepA*, ***yqfDC***, ***yqfZY***, ***yqiQmmgEDCBA***, *yqjXW*, ***yqxAspoIIP***, *yqzG*, ***yrbA***, *yrkC*, *yrzF*, *ysdC*, *ysnD*, *ysnE*, *ytaGFmutMpolA*, *ytcAB*, *yteV*, *ytlQ*, *ytvI*, *ytxC*, *yugO*, *yuiH*, *yunB*, *yuzC*, *ywcA*, *ywfKpta*, *ywlD*, *ywqFE*, *ywrJcotB*, *yxeC*, *yxiQ*, *yxjFEDC*, *yyaD*
**σF** (119)	**U**	***bofC***, *enopgmtpipgkgapyvbQ*, ***gerAAABAC***, ***katX***, *lonB*, *miaA*, *relAaptyrvED*, *rpoBCybxFrpsLGfustufAybaCrpsJrplCDWBrpsS-rplVrpsCrplPrpmCrpsQrplNXErpsNHrplFRrpsErpmDrplOsecYadkmapinfArpmJrpsMKrpoArplQybxAybaEFtruArplMrpsIybaJKcwlD*, ***sigG***, *sinI*, *spo0AI*, ***spoIIQ***, ***spoIVB***, *spoVAFVAEVADVACVABVAA* ***sigFspoIIABIIAAdacF***, ***sspE***, *thiA*, ***tlp***, *yabKmfdspoVTyabMNOPQ*, *yerQyefA*, *yetF*, ***yhcNOP***, *yhfW*, *ykvI*, *ylbBCD*, *ynaC*, *yncC*, *yqxAspoIIP* ***gpr***, *yqzG*, *ytfJI*, *ythA*, ***ytkD***, *yuxHyueKJI*, *ywfN*, ***ywhE***, *ywlB* ***spoIIR***
**σG** (95)	**U**	*adhByraED*, *bofC*, *cotK*, *csgAybxH*, *enopgmtpipgkgapyvbQ*, ***gerAAABAC***, ***gerBABBBC***, ***gerD***, *gpr*, *malSytnP*, *sigG*, *splAB*, *spoIVB*, *spoIVCA*, ***spoVAFVAEVADVACVABVAA*** *sigFspoIIABIIAAdacF*, ***sspB***, ***sspC***, *sspD*, *sspE*, *sspF*, *yabKmfd* ***spoVT*** *yabMNOPQ*, *ybaKcwlD*, ***ycxEgdh***, *ydbI*, *yfhD*, *yfhS*, *yfjQ*, *yfjS*, *yfjU*, ***yhcN*** *OP*, *yhcQ*, *yhfM*, *yhjGH*, *ykjAykkA*, *ykoG*, *ykuJKykzF*, *ykuV*, *ykvV*, *ylaJ*, *ylaM*, *ymfJ*, *ypeB* ***sleB***, *yqfS*, *yqzG*, *ysfA*, *yteJIsspA*, *ythA*, *yvsH*, *ywfBA*, *ywfI*, *ywhE*, *yxjA*
**σH** (279)	**U**	*accAyttI*, *araQP*, ***ccdA***, ***citG***, *codY*, *dal*, *deaDyxiMLKJIyxzGyxiHGyxzCyxiFyxxG*, *dgkAyqfG*, *divIC*, ***ftsA***, ***glgPAD***, *glnA*, ***gltP***, ***kinA***, ***lytE***, ***minDC***, *mtrAhbs*, *opuABA*, *panCB*, ***phrC***, ***phrE***, ***phrF***, ***phrG***, ***phrK***, *pksJKLMNP*, *ppsCBA* ***pbpyoxAyoeA***, *proJH*, *rapG*, *rplWBrpsSrplVrpsCrplPrpmCrpsQrplNXErpsNHrplFRrpsErpmDrplOsecYadkmapinfArpmJrpsMKrpoArplQ-ybxAybaEFtruArplMrpsIybaJKcwlD*, *rtp*, ***sigAdnaG***, ***spo0AyqxM***, ***spo0F,*** *spoVAFVAEVADVACVABVAA* ***sigFspoIIABIIAA***, ***spoVG*** *gcaDprs*, ***spoVStdh*** *kblymcBA*, *srfACAycxA*, ***ureCBA***, ***vpr***, *yabPQ*, *yclF*, ***ycsN***, *ydgE*, *ydhG*, *ydjO*, ***yesLMNOPQRSTUVWXYZyetA*** *lplABCDyetF*, *yfhD*, *yfjDC*, ***ygaI***, *ygxA*, ***yhaR***, *yhbI*, ***yhcR***, *yheM*, ***yisK***, ***yisT***, *yjmABCDEFGHIJ*, *ykgA*, *ykkAB*, *yknYZ*, ***ykoM***, ***ykrQ*** *dat*, *ylmDEF*, ***ymaH*** *ymzC*, ***ynaD***, *yneF*, *yngL*, ***yobO***, *yocKLM*, *yodL*, ***yojL***, *yonV*, *yoqDCBA*, *yosTS*, *yotKJIH*, *yozNyocN*, *yphPilvDypgRQ* ***bsaA***, ***ypiB***, *ypmTSRQ*, *yppD*, *yqcG*, *yqeB*, *yqgY*, *yqiG*, *yqiQmmgE*, *yqjBA*, *yrpD*, ***yrvJ***, *yteVUTSR*, *ytiBA*, ***yttP***, ***ytxJHG***, *yusJKLM*, ***yuxI*** *yukJ*, ***yuxL***, *yuxO*, *yvaX*, ***yveRQPONMLK***, *yvqHI*, ***yvyD***, ***ywfF***, *ywhG*, *ywkBAtdkrpmErhoywjImurZywjHfbaA*, *yxiB*, *yycR*
**σK** (117)	**U**	*atpCDGAH*, *bglS*, ***cgeAB***, ***cgeEDC***, ***cotA***, ***cotC***, ***cotD***, ***cotE***, ***cotF***, ***cotG***, ***cotH***, ***cotM***, ***cotT***, ***cotZY***, ***cwlC***, *fliQR*, ***ftsY***, *gbsBA*, *ggaB*, *pgi*, *pksSymzBymaE*, *racEysmB* ***gerE***, *ribH*, ***spoIVCB***, ***spoIVFBI***, *spoVEmurGBdivIB*, ***spoVFAVFBasddapGA*** *ymfA*, ***spoVK***, *spsKJIGF*, ***yabG***, *ybdBD*, *yceFG*, ***yfhP***, *yfnHGFED*, *yhcP*, *yisCDEFGHI*, *yjgCD*, *yloCDHI*, ***yoaN***, ***yobW***, *yodHI*, *yonU*, *ypbH*, ***yqeE***, *yqzDC*, ***yrbBA***, *ytlABCD*, *ytsD*, *ytxOcotSytxN*, *yueG*, *yugNM*, ***yurS***, *yuzE*, *yvcSR*, *yvdO*, *yvfH*, *ywqM*, *ywrJ* ***cotB***, *yxbDC*
**σL** (39)	**U**	***acoABCL*** *RyfjU*, *bfmBBBABBAA* ***yqiVUTS***, *fnrnarKargS*, ***rocFED***, ***sacClevGFED***, *yuxHyueKJ*, ***yweB***, *ywfEDCBA* ***rocCBA***, *ywiE*
**σM** (10)	**U**	*divIC*, ***yhdKLM***, *ypuA*, *yrhJIH*, *ywhB*, *ywoA*
**σW** (77)	**U**	***abh***, ***divICyabR***, *pbpX*, ***sigWybbM***, *xpaCyaaN*, ***ybfOP***, *ycdB*, ***yceCDEFGH***, ***ydbST***, *ydcB*, ***ydjF***, ***ydjOPyeaA***, *yebC*, *yfhLM*, ***yjbCD***, ***yjoB*** *rapA*, ***yknWXYZ***, ***yndN***, ***yoaF***, ***yoaG***, ***yob I J***, *yozO*, *yqfDC* ***yqfBAyqeZ***, *yqgZ*, *yrhH*, *ysdByscB*, ***yteJI***, *ythB*, *ythQ* ***ythP***, ***yuaIGF***, *yveGF* ***racXpbpE***, ***yvlDCBA***, ***ywaC, ywbN, ywnJ, ywoA, ywrE, yxjI, yxzE***
**σX** (58)	**U**	***abh***, ***csbB*** *yfhO*, *degAyisS*, ***divIC***, ***dltABCDE***, ***lytR***, ***pbpX***, ***pssAybfMpsd*** *ybfN*, ***rapD***, *slp*, ***yceCDEFGH***, *ycgQR*, *ydaJKLMN*, ***ydcB***, ***yjbCD***, *yngC*, *yobIJ*, *yppBponA*, *ypuA*, ***ypuNsigX***, ***yrhH***, *yrvEDCsecDF*, *ytnAasnB*, ***ywbN***, ***ywnJ***, ***ywoA***, *ywtFE*, *yxeED*

Continued

TF[a]		Identified transcption units[b]
**AraR**	**U**	gpsAyphC, rpoArplQ, yeeABC, yoaJ, yorZ, ypuIHG
(30)	**D**	**araE** , **araR** , prsAyhaK, **xsa** , ysfEcstA **abfAaraQPNMLDBAabnA** , yydK
**CcpC**	**U**	degUS, ggaBA, grlBA, yjcM, **ykuM** , ykwC, ynzFG, yorWV
(26)	**D**	**citB** yneN, **citZ** , mraYmurDspoVEmurG, spo0B, ykrL, yndEF, yurL, yybF
**ComK** (116)	**U**	**addBA** , bofCcsbX, **comC** , **comECEBEA** , **comGGGFGEGDGCGBGA** , **comK** , **cspB** , **glcRywpH** , **hipO** , levGFED, **medyjzA** , **ninnucA** , **nrgAB** , **pta** , **rapH** , **recA** , rplBrpsS, **rpsFyyaF** , **smf** , **topA** , xpaCyaaN, ybdBD, **ybdK** , **ycbL** , **yhjB** , yhxD, **yhzC** , **yjbF** yjbG, yjdA, ylnF, **yndG** , **ynzC** , yomK, yozO, yqgML, **yqzG** , **ysxA** , yvcT, **yvrP** , yweA, **ywfL** , ywnJspoIIQ, **ywzA** , **yyaAgidBAthdF**
	**D**	aroHBFcheR, atpEB, citG, dnaXyaaK, dppEykfABC, purT, **rok** , sdhA, sigA, spo0A, yddRS, ydeM, yhjR, ykuJK, ykvJK, ylbBCD, ypfD, yqgQP, yrhJI, ysxD, ytgBA, ytpI, yvdS, ywnH, yyaJ
**CtsR** (68)	**U**	cotN, **ctsRyacHIclpCsmsyacK** LM, gltBA, motBA **clpE** , tlpBmcpAtlpAmcpB, yhcP, ykcBC, yolJIsunTA, yopN, yorSR, yqzC, yviFEflgLKyvyGflgMyvyF, ywjC, yycJI
	**D**	**clpP** , motAclpE, ndhFybcC, spoVStdhkblymcBA, ykhAhmp, yloIpriA, ynbBglnR, yosDC, yraFadhB, yrbC, yulEDCByuxG, yuxLthrB, yvcBA
**Fur** (135)	**U**	ccdA, dnaGyqxD, gcaDprs, metBypfP, **nasFE** , pdhBCD, pdpnupCdra, ybaRS, ycdHIyceA, ycgJ, ycgRS, ydbL, ydhMNOPQRST, **ydhU** , yfnA, yjdA, ymcBA, yncB, yptA, yqfON, yuzC, yvbTU, ywhG, yxnBasnH, yydD, yydJ
	**D**	ahpCF, fhuCGB, **fhuD** , hemLBDCXA, mrgA, tig, **ybbAfeuCBAybbB** , **ybbBCDEFHIJK** , ycgT, yclNOPQ, ydbN, **yfhC** , yfiY, yfiZyfhA, yfjR, **yfkM** , yfmFEDC, **yhfQ** , **ykuNOP** , ykvW, **yoaJ** , yolKJIsunTA, yorJIHGFE, yppQP, ytsP, **yuiI** , **yukLMdhbFBECA** , yumC, yurUVWXY, **yusV** , yvgZ, **ywbONML** , **ywjBA** , **yxeB**
**GlnR**	**U**	**glnRA** , spoIVCA, yclJK, yexApurLQFMNHD, yhcGHI, yqhN, yrkLK, yyaC, yybMLKJ
(53)	**D**	alsT, **nasA** , **nasB** , **nasC** , **nasFED** , nrgA, tnrA, **ureCBA** , xkdNO, ykzBykoL, yoaZ, yqjED, yqkKJ, ytpB, yunGH, yunIJKLM
**HrcA**	**U**	**yqeVUTdnaJKgrpEhrcA** , yurXY
(19)	**D**	**groESEL** , ybaRS, ydiKL, yerQyefA, ytqAB
**IolR** (32)	**U**	cmk, yjbCD, yndB, yobJ, yokFE, ypiBA, yybNMLKJ
	**D**	ahpCF, **iolRS** , msmRE, pdpnupCdradeoR, proSpolC, ydhOP, **ydjK** , yojA, ywfBA
**LmrA**	**U**	**lmrBA**
(7)	**D**	yfkCBA, **yxaHG**
**PerR** (127)	**U**	citAyhdF, dacAyaaDE, murEmraYmurDspoVEmurGBdivIBylxW, oppABCDFyjbB, sigWybbM, ybdKL, yclNOPQ, yluAcdsAyluBCproSpolCylxSnusAylxRQinfBylxPrbfAtruBribCrpsOpnpAylxYymxG, ynzD, yoaEF, yolKJIsunTA, yorGF, ytcFgapB, yueCByukABC, yusA, ywjA, yxeB, yxkC
	**D**	**ahpCF** , citR, gltBA, **hemLBDCXA** , hemY, **katA** , **mrgA** , murEmraYmurDspoVEmurGBdivIBylxWXsbp, ppsCBApbpyoxAyoeA, yaaH, ydbO, ydeLM, yfkM, yfmJ, **ygaG** , **ykvW** , ylmAB, yncB, yobL, yokJI, yozIyobE, yphPilvD, yppQ, **yqkL** , yrhED, yrhF, yukLMdhbFBECA
**PurR**	**U**	clpCsms, flhPO, infArpmJ, lrpA, nusAylxRQinfB, **purRyabJ** , rplErpsN, yacBCD, ycgFG, ydeST, ykuG, ylqFrnhylqGH, yopJI,
(81)	**D**	appDFAB, atpBIupp **glyA** , gltP, **pbuXxpt** , ppsApbpyoxAyoeA, **purA,** **purEKBCyexApurLQFMNHD** , recNahrCyqxCyqiEDCBfolD **yqhZ** , yaaDE, ydaR, **yebB** , yfjBA, yjdJ, ykaAykbA, **ytiP** , **yumD**
**ResD** (159)	**U**	codY, **ctaA** , cysH, deaDyxiMLKJIyxzGyxiHGyxzCyxiFyxxGwapAyxxFyxiE, **fnr** , hemLBDCXA, **hmp** , **nasFED** , smbAfrryluAcdsAyluBCproSpolCylxSnusAylxRQinfBylxPrbfAtruBribCrpsOpnpAylxYymxG, ybbAfeuCBAybbB, **yclJ** , yeeAB, yerQyefA, yjdB, yobB, yocE, ypuI, yqgS, yraON, yrkON, ytbQbioIBDFAW, yttB, ytzFGytgP, yvbT, yxaLK
	**D**	adaB, bprspoIIGAsigEG, ccdA, dppABCDEykfABCD, odhBA, oppAB, rocFED, spoIIEyabST, spoVStdh, ungywdF, ybbEFHIJK, ybxG, yclM, yczG, ydcMN, yddT, yeeAB, yfnA, yhjGH, yitK, yknA, ylbA, yomL, yosQP, yrhG, yrhJI, ysnBA, yurL, yutJ, yvaDEF, ywbBA, yweA ywfCBArocCBA, ywhA, yxaBA, yybP, yydC
**RocR**	**U**	**rocFED** , **rocR** , rpmErhoywjImurZywjHfbaA, trpFC, ycgJ, yomK, ywfCBA **rocCBA** , ywiEnarIJHG
(39)	**D**	appBCyjbA, bglA, comGGGF, fhuD, oppBCDFyjbB, tdkrpmE, ybgHJ, yitM, yodPargE, ytcQ
**SinR** (95)	**U**	cheV, fliIJylxFfliKylxGflgEfliLMYcheYfliZPQRflhBAFylxHcheBAWCDsigDylxLrpsB, lytCBA, odhBA, sucCD, tlpBmcpAtlpAmcpB, wprA, yaaDE, yopR, yqiDCBfolD, yrkA
	**D**	**aprE** , **comK** , **cotNsipWyqxM** , glcRywpH, kinB, **sigFspoIIABI** , slr, **spo0A** , **spoIIGAsigEG** , spoIIIJrnpA, spsKJIG, yesOP, yfmPO, ykrL, **ykuW** , yrdBA, ytgDC, yurK, **yveTSRQPONMLK** , yvfEDCBA
**TreR**	**U**	atpIupp, pksS, ypfBA
(30)	**D**	araNMLD, csn, murFydbRST, narA, pyrDF, tagHG, **trePAR** yfkO, xkdKM, yfkRQ, yhdI, yqjTS
**XylR**	**U**	sinI, xylR, yceCDEF, ygaE, yocJ, yqiYX, yviEflgLKyvyG
(54)	**D**	mtlAD, pyrRP, xtmBxkdEFG, **xylAB** , **xynB** , **xynP** , ycgLM, ydaJK, yfnHG, yhaGserC, yhfEF, ykrTU, ymfFGHIJ, ytxEDccpA, yulByuxG, yusKL, yvaN, ywdJI
**Zur** (54)	**U**	amhX, divIC, dps, yabFksgA, yceI, yefB, yfiO, yloW, yolJIsunTA, yomL, yopKJIHGFEDC, yqcKJ, yuxOcomA, yybKJ
	**D**	brnQazlDCB, citR, ctrArpoE, **ycdHIyceA** , **yciABC** , yfhC, yhzA, yodDE, yraO, yrpE, ytiBA, yuxOcomA, yvgQR

[a]**U** and **D** refer to genes that are up- and down-regulated, respectively, in the presence of the TF. Figures in parentheses indicate number of genes regulated by the TFs.

[b]All data were identified under 1% threshold in PWM and 5% threshold in deletion mutant array with FDR. Among these genes, the known genes regulated by the TF are indicated in bold.
