# Supplementary figures and images for: The entire organization of transcription units on the Bacillus subtilis genome
Source: BMC Genomics. 2007 Jun 28;8:197. doi: 10.1186/1471-2164-8-197 (PMC1925097; doi:10.1186/1471-2164-8-197)

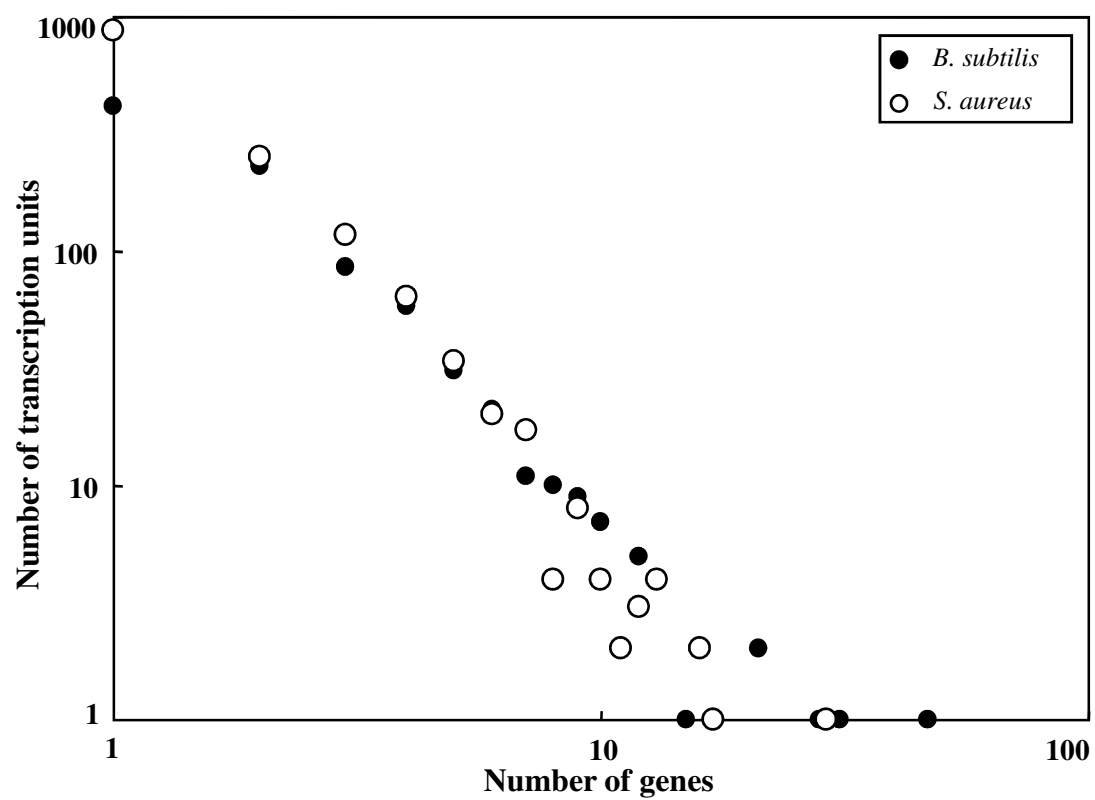

Supplement: Additional file 2 — Supplementary Figure 1. The relationship between the number of TUs detected in the present study and the number of genes composing the TUs. [file 1471-2164-8-197-S2.pdf]
